# Association between learning styles and burnout among medical students: A study at Alfaisal University

**DOI:** 10.12669/pjms.42.1.11587

**Published:** 2026-01

**Authors:** Saed Fawaz Raddawi, Jarah Khaled Almutairy, Aya Firas AlMousli, Shoukat Ali Arain, Aamir Omair, Sultan Ayoub Meo

**Affiliations:** 1 Saed Fawaz Raddawi, Medical Student, College of Medicine, Alfaisal University, Riyadh, Saudi Arabia; 2 Jarah Khaled Almutairy, Medical Student, College of Medicine, Alfaisal University, Riyadh, Saudi Arabia; 3 Aya Firas AlMousli, Medical Student, College of Medicine, Alfaisal University, Riyadh, Saudi Arabia; 4 Shoukat Ali Arain, MBBS, PhD Department of Pathology, College of Medicine, Alfaisal University, Riyadh, Saudi Arabia; 5 Aamir Omair, MBBS Department of Medical Education, King Saud bin Abdulaziz University for Health Sciences, Riyadh 11426, Saudi Arabia; 6 Sultan Ayoub Meo, MBBS, PhD Department of Physiology, College of Medicine, King Saud University, Riyadh, Saudi Arabia

**Keywords:** Burnout, Collaborative, Disengagement, Emotional Exhaustion, Learning styles, Medical education, Medical students, Saudi Arabia

## Abstract

**Objectives:**

This study investigated the relationship between learning styles and burnout among undergraduate medical students.

**Methodology:**

This cross-sectional survey was conducted at Alfaisal University, Riyadh, Saudi Arabia, between April and June 2024. Burnout levels were assessed using the Oldenburg Burnout Inventory, and learning style preferences were assessed using the Grasha Reichmann Learning Style Scale. Demographic information, including age, gender, academic year, and living situation, was also collected. Multiple linear regression (MLR) was used to identify independent predictors of burnout components, controlling for gender.

**Results:**

A total of 161 students completed the questionnaire. Females had significantly higher exhaustion scores than males. Males preferred independent and competitive learning styles. The MLR analysis revealed that the avoidant learning style was the strongest predictor of total burnout (β = 3.14), indicating that disengagement is the primary driver of burnout. Conversely, the collaborative style significantly buffered against emotional detachment (β = -1.32), and the participant style significantly reduced emotional exhaustion (β = -1.89). The MLR confirmed that the relationship between avoidant style and burnout was independent of gender. Dependent, independent, and competitive learning styles had no significant predictive impact on burnout. Also, the study found no link between age, living situation, and learning style preferences.

**Conclusions:**

The avoidant learning style is an independent risk factor for burnout in undergraduate medical students, while collaborative and participant styles are protective. The findings emphasize the importance of early screening for avoidant characteristics and using adaptable teaching techniques that promote engagement and teamwork to mitigate high-risk learning behaviors and reduce burnout rates. Future work could investigate the mechanisms underlying this relationship and the roles of other variables, such as social support and personality traits.

## INTRODUCTION

Burnout is a psychological syndrome resulting from chronic emotional stress associated with a particular job.^1^ In the context of students, it refers to the exhaustion brought by demanding studying, leading to ineffectiveness and low efficiency.^2^ It can hinder the personal and professional development of medical students. Importantly, physicians’ well-being could also be vital to the delivery of high-quality health care. Burnout can lead to medical errors, lower patient satisfaction, and overall poorer quality of healthcare.^3^

Medical students may experience declining well-being due to stress as early as their first year of training.^4^ Similarly, moderate burnout is prevalent among postgraduate residents and junior consultants working in tertiary care settings.^5^ A meta-analysis of global studies on burnout using data from 42 studies involving over 26,000 medical students reported a prevalence of nearly 37%.^6^ Another systemic review found higher burnout levels in medical students compared to the general population in China.^7^ Similarly, Saudi Arabian studies reported the burnout prevalence between 13.4% to 67%.^8^,^9^ This study employed the Oldenburg Burnout Inventory (OLBI), a 16-item self-report questionnaire. The OLBI assesses exhaustion and disengagement, with higher scores indicating higher levels of burnout.^10^ OLBI has been shown to have good reliability and validity, with both positive and negative phrased items. Moreover, OLBI has a more specific focus on healthcare settings.^11^

While variations in the assessment tools and cut-off scores used across studies can contribute to inconsistencies in reported burnout prevalence, society-specific social factors, including family structure and lifestyle choices, personality traits, and the specific characteristics of the medical school learning environment, also play a role in these differences.^12^,^13^ Academic performance, extracurricular involvement, and learning styles can also contribute to burnout among medical students.^14^ Learning styles include cognitive, physiological, and emotional characteristics that determine how the learner understands and reacts to the learning environment.^15^ We employed the Grasha-Reichmann Learning Style Scale (GRLSS). The GRLSS categorizes learners into six styles. Independent learners are self-directed, while avoidant learners may disengage from academic activities. Collaborative learners value teamwork and knowledge sharing, whereas dependent learners seek guidance. Competitive learners thrive on outperforming peers, and participant learners prefer hands-on experiences.^16^

By understanding the relationship between learning styles and burnout, we can tailor educational approaches to enhance student engagement and reduce burnout. Thus, our study aimed to investigate the relationship between learning styles and burnout among medical students at Alfaisal University, Riyadh, Saudi Arabia. By using the OLBI and the GRLSS, we sought to identify specific learning styles associated with higher or lower levels of burnout.

## METHODOLOGY

This cross-sectional study was conducted between April and June 2024 on undergraduate medical students at Alfaisal University. Data were collected via convenience sampling using a questionnaire created in Google Forms. A link was sent to all undergraduate medical students through an official university email. Data from all the students who gave consent and completed the survey were included. All data were anonymised by coding email addresses upon download. The primary research dataset contained only the anonymous response data and is stored on a password-protected, encrypted university drive accessible only to the research team. Also, all results are reported in aggregate. All the data files will be retained for five years and then securely destroyed.

### Ethical Approval:

The study was approved by the Institutional Review Board, Alfaisal University, Riyadh, Saudi Arabia (IRB-20295; dated: April 15, 2024).

### The questionnaire:

The questionnaire consisted of three components. The first component comprised students’ sociodemographic variables, including age, gender, academic year, and students’ living situation (alone, with friends, with family). The second component measured students’ burnout levels using the Oldenburg Burnout Inventory (OLBI). It consisted of 16 items that evaluated burnout across two dimensions —exhaustion and disengagement —each containing eight items. Each item was scored on a 4-point Likert scale, with total scores ranging from 16 to 64. Higher scores indicate higher levels of burnout. The final component assessed students’ learning styles using the Grasha Reichman Learning Style Scale (GRLSS). This scale consisted of 60 items measuring six learning styles (independent, avoidant, collaborative, dependent, competitive, and participant) on a 5-point Likert scale. Each style has ten questions, and the mean score of these questions is then calculated. The internal consistency (Table-I) of the OLBI and GRLSS scales was assessed using Cronbach’s alpha (α). The alpha values ranged from 0.634 (disengagement) to 0.834 (competitive), with three scales falling below the 0.70 threshold (disengagement: α= 0.634; independent: α = 0.691; dependent: α= 0.672).

**Table-I:** Cronbach alpha coefficients for OLBI and GRLSS.

Survey component	Item N	Cronbach’s alpha
Disengagement	8	0.634
Exhaustion	8	0.715
Independent	10	0.691
Avoidant	10	0.751
Collaborative	10	0.797
Dependent	10	0.672
Competitive	10	0.834
Participant	10	0.752

OLBI: Oldenburg Burnout Inventory. GRLSS: Grasha Reichman Learning Style Scale

### Statistical analysis:

The data were coded and analyzed using the Statistical Package for the Social Sciences version 27 (SPSS Inc., Chicago, IL). Descriptive statistics were used to summarize the demographic characteristics of the participants. Burnout dimensions and learning style scores were calculated as described in the original surveys. The association between socio-demographic factors and burnout and learning styles was assessed using independent t-tests and ANOVA. Additionally, we examined the association between learning styles and burnout using multiple linear regression (MLR). Three separate MLR models were constructed, with the continuous scores for total burnout, exhaustion, and disengagement serving as the dependent variables. The independent variables in all models included the continuous scores for the learning styles found to be significant in preliminary bivariate analysis (avoidant, collaborative, and participant). Also, gender was included in all models as a control variable to account for its potential confounding effect. Unstandardized regression coefficients (β), standard errors (SE), adjusted R^2^, and the overall model *F*-statistic were reported for each MLR model. Statistical significance was set a priori at p < 0.05.

## RESULTS

With an enrolment of approximately 600 students, the final sample of 161 represented a response rate of 26.8%. The socio-demographic information (Table-II) shows that most participants (80%) were aged between 18 and 22 years, while 91 (57%) were male. Moreover, 66 (41%) were in the third year, and 128 (80%) lived with their family. The mean total burnout score was 41.1±7.5, with mean scores of 21.7±4.2 for the exhaustion subscale and 19.5±4.0 for the disengagement subscale.

**Table-II:** Summary of the questionnaire data (N=161).

Age groups (in years)	Count (%)
18-22	129 (80)
>22	032 (20)
***Gender***	
Male	91 (57)
Female	70 (43)
***Academic Level***	
Year 1	41 (25)
Year 2	35 (22)
Year 3	66 (41)
Clinical years	19 (12)
***Living situation***	
With family	128 (80)
With friends	011 (07)
Alone	022 (14)
***Learning Style Scores***	***Mean (SD)***
Independent	3.6 (0.6)
Avoidant	3.4 (0.7)
Collaborative	3.5 (0.7)
Dependent	3.6 (0.6)
Competitive	2.7 (0.8)
Participant	3.2 (0.7)
**Burnout Scores**	
Disengagement	19.5 (4.0)
Exhaustion	21.7 (4.2)
Total Burnout score	41.1 (7.5)

The mean scores for the learning styles shows in Table-II. The preferred learning styles were the independent (3.6±0.6) and the dependent (3.6±0.6). The collaborative style (3.5±0.7) and the avoidant style (3.4±0.7) also showed relatively high endorsement. The participant style was rated slightly lower (3.2±0.7), and the competitive style was the least preferred (2.7±0.8). This distribution highlights a population that endorses both autonomous and teacher-led learning.

In Table-III, illustrates the comparison between burnout and demographic factors. There was no significant difference in disengagement, exhaustion, or total burnout scores among participants across age groups, academic years, or living situations. However, females yielded significantly higher mean exhaustion scores than males (22.7±3.7 vs 21.1±4.2; p=0.009).

**Table-III:** Comparison of Burnout Subscale Scores and Total Burnout Score Among Medical Students: A Demographic and Academic Level Analysis.

	Disengagement		Exhaustion		Total Burnout	
	Mean (SD)	p-value	Mean (SD)	p-value	Mean (SD)	p-value
Age groups	18-22 yrs.	19.5 (3.7)	0.529	21.8 (4.0)	0.673	41.3 (6.9)	0.922
	>22 yrs.	19.9 (4.1)		21.5 (4.1)		41.5 (7.2)	
Gender	Male	19.5 (3.6)	0.679	21.1 (4.2)	0.009*	40.5 (7.0)	0.085
	Female	19.7 (4.0)		22.7 (3.7)		42.4 (6.9)	
Academic level	Year 1	19.5 (3.5)	0.614	22.0 (3.7)	0.853	41.5 (6.4)	0.901
	Year 2	19.1 (4.3)		22.1 (4.5)		41.2 (8.1)	
	Year 3	19.6 (3.6)		21.5 (4.1)		41.1 (6.9)	
	Clinical years	20.5 (4.1)		21.9 (4.1)		42.4 (7.0)	
Living status	With family	19.6 (3.9)	0.790	21.9 (4.1)	0.699	41.6 (7.1)	0.720
	With friends	19.7 (4.3)		21.4 (3.8)		41.1 (7.1)	
	Alone	19.1 (3.2)		21.2 (3.8)		40.3 (6.5)	

*Significant difference at p<0.05. p-values were determined using independent samples t-test and ANOVA.

**Fig.1:**
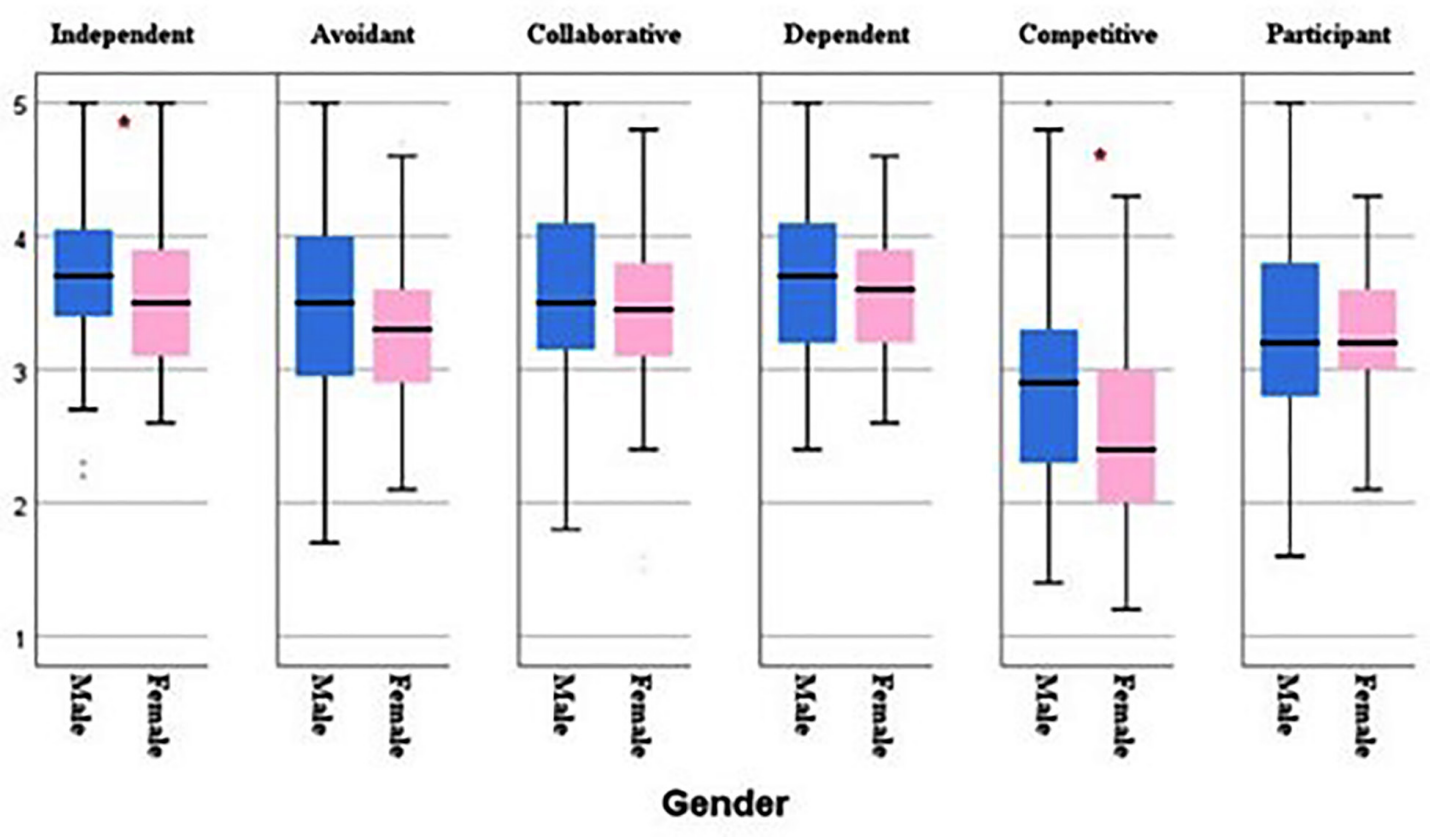
Gender-based analysis for learning style preferences. *Significant difference at p<0.05 (males preferred these learning styles).

As illustrated in Fig.1, males demonstrated a stronger preference for independent (3.72 ±0.6 vs 3.53±0.5; p=0.031) and competitive (2.89±0.8 vs 2.497±0.8; p=0.002) learning approaches than females. However, no other demographic factors (age, academic year, living situation) were associated with significant differences in learning styles.

Table-IV illustrates the multiple linear regression analysis of learning styles predicting burnout dimensions. The regression model explained 26% of the variance, with the avoidant learning style showing a positive association (β = 3.14), while the collaborative (β = -1.87) and participant (β = -2.45) styles showed negative associations with burnout. The overall model explained 18% of the variance, with avoidant learning style showing a positive association (β = 1.98) and participant learning style showing a negative association (β = -1.89) with exhaustion. These two learning styles were significantly associated with disengagement, accounting for 19% of the model’s overall explanation; the avoidant learning style had a positive association (β = 1.16), while the collaborative learning style had a negative association (β = -1.32). After controlling for the effects of all three learning styles, female gender remained an independent, positive, and significant predictor of the exhaustion subscale (β = 1.95). Gender was not a significant predictor of total burnout or disengagement in these models.

**Table-IV:** Multiple Linear Regression Analysis of Learning Styles Predicting Burnout Dimensions (Controlling for Gender)

Variable	Total Burnout	Exhaustion	Disengagement
Model Statistics	β (SE)	β (SE)	β (SE)
***Learning Styles Scores***			
Avoidant	3.14*** (0.55)	1.98*** (0.42)	1.16** (0.55)
Collaborative	-1.87** (0.49)	-0.55 (0.37)	-1.32*** (0.33)
Participant	-2.45*** (0.61)	-1.89*** (0.46)	-0.56 (0.41)
***Demographics (Control Variable)***			
Female (ref: Male)	1.58 (0.78)	1.95* (0.59)	-0.37 (0.52)
***Model Summary***			
R^2^ (Adjusted)	0.26	0.18	0.19
F-statistic	9.45	6.22	6.71
p-value	<0.001	<0.001	<0.001

β: Unstandardized regression coefficient. (SE) Standard Error.

*** p<0.001,

** p<0.01,

* p<0.05

## DISCUSSION

This study assessed the relationship between learning styles and burnout among medical students. The findings revealed that females had significantly higher exhaustion scores than males, while males preferred independent and competitive learning styles compared to females. Importantly, collaborative and participant learning styles were associated with lower levels of burnout. In contrast, avoidant learning styles were linked to higher levels of burnout. Dependent, independent, and competitive learning styles were not associated with burnout. This study showed that females had significantly higher exhaustion scores than males. The MLR analysis also confirmed that the higher level of exhaustion experienced by female students is independent of their learning style preferences. This finding is consistent with previous studies.^8^,^17^,^18^ According to Altannir et al., cultural, social, and religious factors may contribute to the higher exhaustion scores observed among female medical students in Saudi Arabia.^8^

The findings of this study also indicated that males had significantly higher mean scores in the independent and competitive learning styles. Our findings align with previous research indicating that males exhibit a higher independent learning style. However, in the same study, males also scored higher in the avoidant learning style, suggesting a complex interplay of factors influencing learning style.^18^ Similarly, another study found that males had a higher mean score for the independent and avoidant learning styles, while females had higher scores for collaborative, participative, and dependent styles.^16^ We are unsure about the exact cause of higher independent and competitive learning style scores in our cohort; however, we hypothesize that societal expectations and stereotypes about traditional gender roles might influence how individuals learn; males are encouraged to be independent and assertive. Besides, different educational experiences before starting university. Exposure to different teaching styles or curriculum content can also impact learning styles.^19^

The MLR analysis provides definitive evidence of predictive relationships between learning styles and burnout dimensions. A high beta coefficient for the avoidant learning style (β= 3.14) establishes it as the single most potent driver of burnout in this cohort of undergraduate medical students. This finding is consistent with the conceptual link between disengagement and burnout, suggesting that students who avoid academic activities are at the highest risk for developing burnout.^20^

On the other hand, the collaborative style (β=-1.32) emerged as a protective factor specifically mitigating feelings of emotional detachment. Also, the participant style (β=-1.89) protected emotional exhaustion. This indicates that active, hands-on, and engaging learning buffers for the overwhelming sensation associated with the demanding workload inherent to medical education. The lower levels of burnout associated with these constructive learning styles support the theory that social support may help prevent burnout.^21^,^22^ The findings corroborate recent studies that highlight the prevalence of high-demand, stressful academic environments in medical schools and underscore the need for student-driven coping mechanisms, such as peer support, to mitigate systemic vulnerability to burnout.^23^,^24^

The significant association between learning styles and burnout has several important implications. Educators should adapt diverse learning strategies, like opportunities for collaboration and independent study, to cater to diverse learning preferences. Identifying avoidant learners at an early stage is also essential to develop and implement coping strategies to prevent burnout. These strategies might include personalized academic support, peer support groups, professional counselling services, or adjustments to course workload.

Apart from gender, we did not find an association between other demographic factors (age, living situation) and learning style preferences. This lack of association could be attributed to the demographic composition of our sample, which primarily consisted of individuals aged 18-22 years (80%) living with their families (80%). Also, in contrast to earlier studies,^19^ our analysis revealed no significant association between academic year and learning style preferences, as only 12% of our participants belonged to clinical years. The limited variation in these factors may have reduced our ability to identify significant differences.

### Limitations

Firstly, data were collected via an online questionnaire, which may have introduced response bias because participants self-selected. Secondly, the cross-sectional design limits our ability to determine causation between learning modalities and burnout. An important limitation of the study is its relatively small sample size; this may be due to the survey’s length, which may have deterred some potential participants. Also, the participants were from a single university.

Larger-scale studies would strengthen the generalizability of the results, while longitudinal studies are essential to establish the causal relationship between learning styles and burnout.

A crucial methodological limitation was the marginally low internal consistency observed for the disengagement (α = 0.634), independent (α = 0.691), and dependent (α = 0.672) scales. While these values are acceptable for exploratory research, they suggest potential issues with the item homogeneity or cultural specificity of these measures in a Saudi medical context. Consequently, the findings related to these three scales should be interpreted with caution.

## CONCLUSIONS

This study identifies specific learning styles as independent predictors of burnout among undergraduate medical students. The avoidant learning style emerged as an essential and independent risk factor for burnout. Conversely, the collaborative and participant styles function as significant protective factors against disengagement and emotional exhaustion, respectively. Beyond learning style, this research confirms two key demographic findings: a significant difference in preference, with males favoring independent and competitive learning, and a notable vulnerability, with females reporting significantly higher emotional exhaustion scores.

These results emphasize the importance of using adaptable teaching methods and curriculum design that discourage avoidant learning behaviors and promote collaborative and participant engagement to reduce burnout rates effectively. To provide a more comprehensive picture, further research could explore the mechanisms underlying the relationship between learning style and burnout, as well as the roles of other variables, such as social support and personality traits.

### Author`s Contribution:

**SFR, JAK, and SAA** conceived the study.

**SFR, JAK, and AFA** conducted data acquisition.

**AFA, SAA, and AA** performed the analysis.

**SAA, AA, and SAM** wrote the initial draft.

All authors reviewed, revised, and approved the final manuscript.
